# Case Report: Flurbiprofen-induced Type I Kounis syndrome

**DOI:** 10.3389/fcvm.2023.1284408

**Published:** 2023-11-20

**Authors:** Chao Tang, Yuqi Chen, Xiaosong Gu

**Affiliations:** Department of Cardiology, The Second Affiliated Hospital of Soochow University, Suzhou, China

**Keywords:** Kounis syndrome, allergy, acute myocardial infarction, flurbiprofen, case

## Abstract

**Background:**

Kounis syndrome is a specific type of acute coronary syndrome caused by allergic or hypersensitivity response. Clinical knowledge about this syndrome is insufficient. We report a case in which intravenous administration of flurbiprofen resulted in Type I Kounis syndrome.

**Case summary:**

A 60-year-old female patient with no history of coronary artery disease developed limb erythema, hypotension, and chest tightness after receiving intravenous flurbiprofen. Electrocardiogram showed ST segment elevation in leads II, III, and aVF. Emergency coronary angiography revealed no significant stenosis or thrombus in the coronary arteries. Subsequent echocardiography showed no apparent abnormalities. Levels of troponin T were elevated. The diagnosis was flurbiprofen-induced Type I Kounis syndrome, presenting as acute ST segment elevation myocardial infarction.

**Conclusions:**

Patients with Kounis syndrome can exhibit severe clinical symptoms, and their condition may even be life-threatening. It is important for clinicians to have a thorough understanding of this syndrome in order to develop comprehensive treatment plans.

## Introduction

Kounis syndrome is defined as a type of acute coronary syndrome associated with the activation of various inflammatory mediators and cytokines caused by allergic reactions and the activation of mast cells and platelets (1). Myocardial injury is common in allergic patients; however, many cases of Kounis syndrome may go undiagnosed due to atypical symptoms and a lack of awareness among clinical doctors (2). Additionally, current treatment options are not standardized. In this report, we present a case of Kounis syndrome induced by flurbiprofen.

## Case presentation

A 60-year-old female patient presented to our emergency department on June 12th, 2023 with a complaint of upper abdominal pain persisting for the past two weeks. Abdominal ultrasound in an external hospital revealed cholecystitis with sandy gallbladder stones. The patient had a 10-year history of hypertension and had been managing her asthma for more than 4 years with appropriate treatment. Additionally, the patient had experienced a rash-like allergic reaction when previously using omeprazole and rabeprazole injection solutions.

In the emergency room, the patient was treated with fluids. After the first dose of intravenous infusion of flurbiprofen ester was completed, the patient developed symptoms including cold sweats, chest tightness, shortness of breath, fatigue, red rashes on the trunk, accompanied by itching, mainly on the lower extremities. This was followed by prolonged chest pain, described as dull, and approximately 15 min later, then the patient experienced confusion and collapsed to the ground. She was immediately transferred to the resuscitation room for treatment. Upon arrival in the resuscitation room, the patient was conscious with a blood pressure of 75/49 mmHg, a heart rate of 58 bpm, peripheral oxygen saturation around 80%, and tenderness in the umbilical area. The patient was treated with intravenous administration of diluted methylprednisolone 40 mg, intravenous administration of dexamethasone 5 mg, subcutaneous injection of epinephrine 0.5 mg, and intravenous drip of metaraminol bitartrate injection.

**Figure 1:**
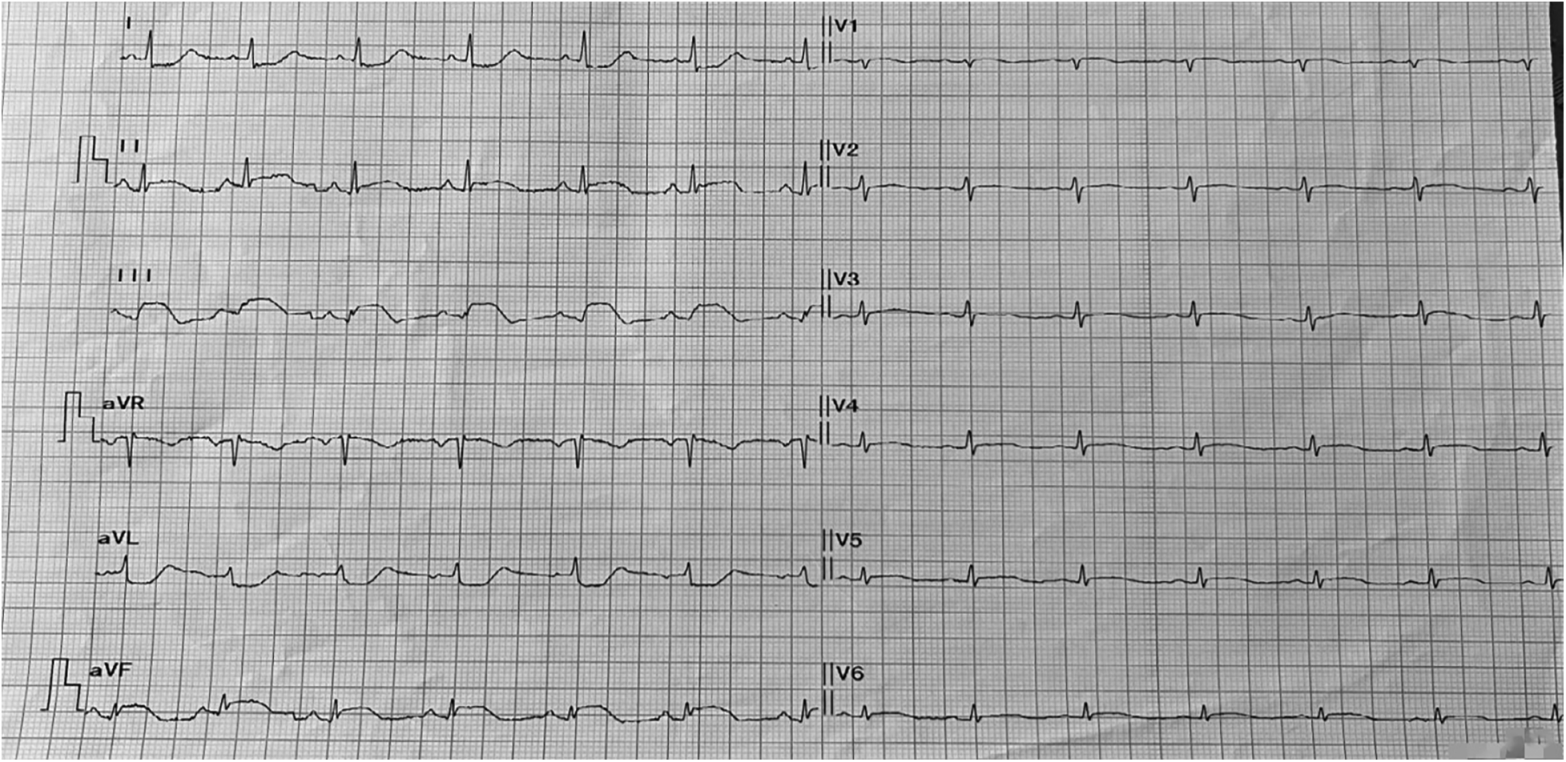
The 12-lead electrocardiogram in patient with chest tightness and discomfort showed ST segment elevation in leads II, III, and aVF.

**Figure 2:**
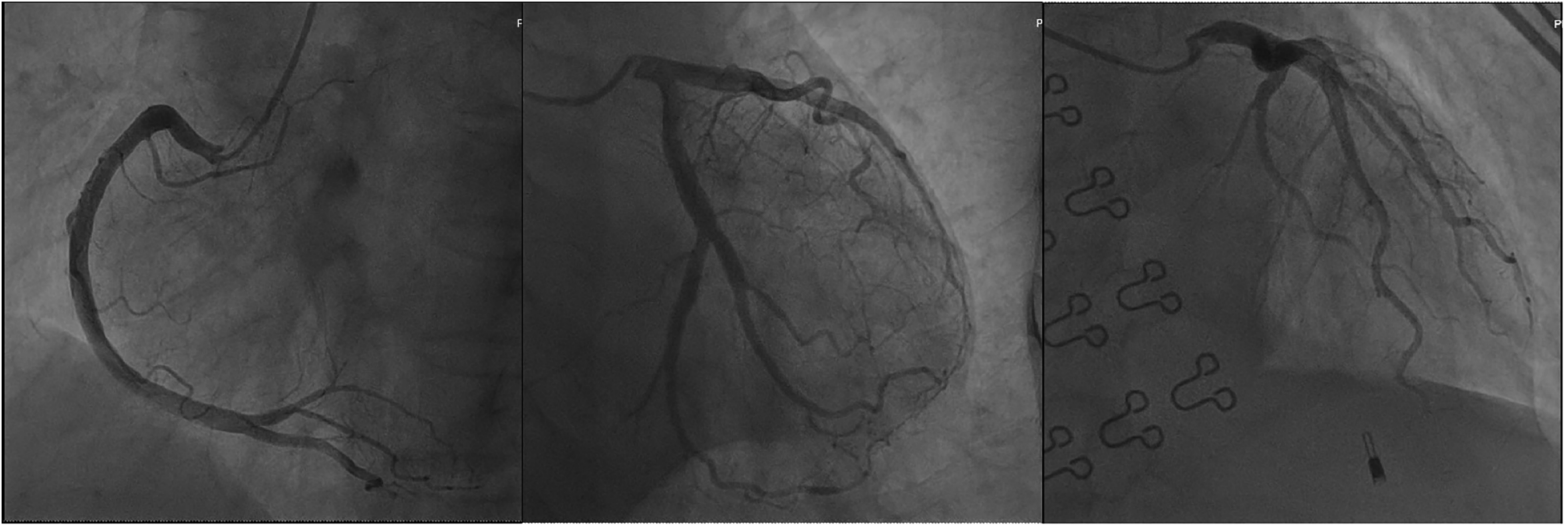
Coronary angiography revealed no significant coronary artery stenosis or thrombus formation.

**Figure 3:**
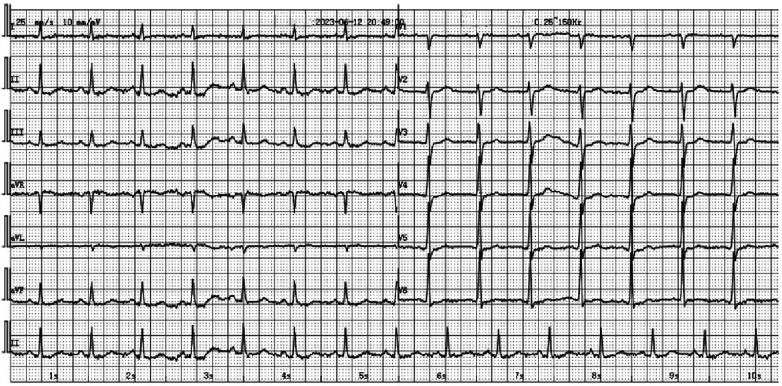
The 12-lead electrocardiogram in patient after coronary angiography showed the disappearance of ST segment elevation in leads II, III, and aVF.

An electrocardiogram showed ST segment elevation of 0.2–0.3 mV in leads II, III, and aVF, and depression of 0.1–0.2 mV in leads I and aVL (Figure 1). Arterial blood gas analysis showed a pH of 7.39, a partial pressure of carbon dioxide of 38.0 mmHg, a partial pressure of oxygen of 42.0 mmHg, an oxygen saturation of 72.1%, and a lactate level of 1.7 mmol/L. The white blood cell count was 6.0 × 10^9^/L (normal range: 3.5–9.5 × 10^9^/L), lymphocyte count was 2.5 × 10^9^/L (normal range: 1.1–3.2 × 10^9^/L), eosinophil count was 0.39 × 10^9^/L (normal range: 0.02–0.52 × 10^9^/L), and eosinophil percentage was 6.5% (normal range: 0.4%–8.0%). Creatine kinase isoenzyme was 1.88 ng/ml (normal range: <3.77 ng/ml), myoglobin was 25 ng/ml (normal range: 25–58 ng/ml), troponin T was 4 pg/ml (normal range: <30 pg/ml), and N-terminal pro-brain natriuretic peptide (NT-proBNP) was 174 pg/ml (normal range: <300 pg/ml). Liver and kidney function tests and coagulation tests were normal. Acute myocardial infarction was suspected, and the patient was treated with subcutaneous injection of heparin 3,000 units, oral ticagrelor tablets 180 mg, and oral aspirin tablets 300 mg. Emergency coronary angiography showed no significant stenosis or thrombus in the coronary arteries (Figure 2). Thrombolysis in myocardial infarction (TIMI) blood flow grade III was observed. After coronary angiography, the patients' heart rate was maintained at around 80 bpm and blood pressure around 100/60 mmHg. Post-angiography electrocardiography showed sinus rhythm, and the ST segment elevation in the inferior leads decreased (Figure 3). A bedside echocardiogram indicated no obvious segmental wall motion abnormalities in the left ventricle.

**Figure 4:**
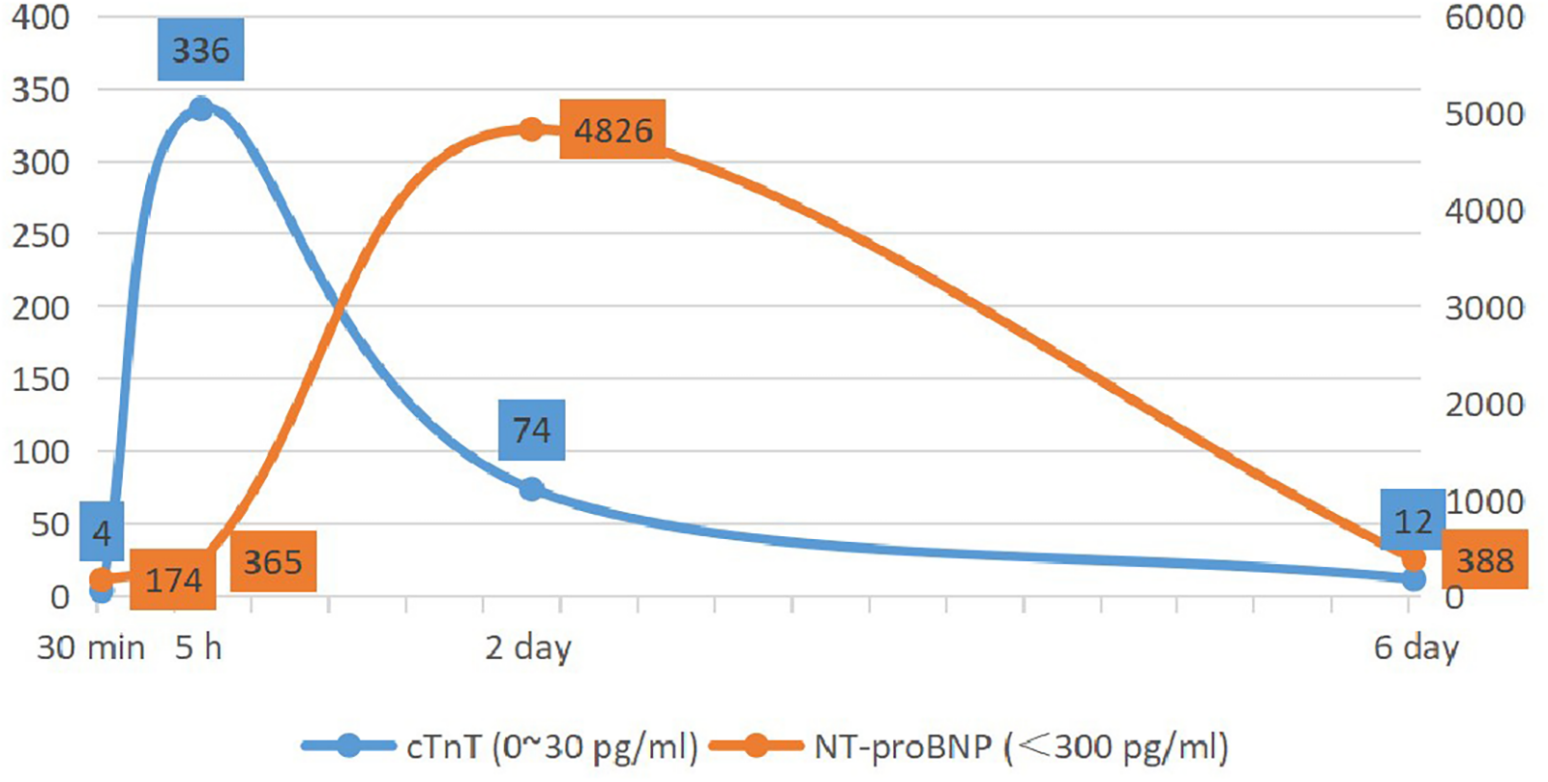
Changes in serum levels of cardiac troponin T (cTnT) and N-terminal pro-brain natriuretic peptide (NT-proBNP).

Based on the patient's medical history, physical symptoms, and relevant laboratory findings, coronary spasm due to flurbiprofen ester allergy (Kounis syndrome) was considered. Post-angiography, the patient was treated with cetirizine 10 mg once daily for 3 days, as well as specific treatments such as hepatobiliary drugs, anti-infection therapy, and myocardial nutrition therapy. On the third day, the patient's NT-proBNP levels significantly increased (Figure 4), and furosemide was administered for diuresis while controlling fluid replacement. Then the patient's condition improved, and she was discharged on the 7th day. The follow-up echocardiogram before discharge showed good motion of all segments of the left ventricular wall, with a left ventricular end-diastolic diameter of 42 mm and an ejection fraction of 65%. At the 2-month follow-up, the patient reported no recurrence of chest tightness or chest pain and expressed satisfaction with the treatment.

## Discussion

Flurbiprofen, as a nonsteroidal anti-inflammatory drug, can selectively inhibit cyclooxygenase, block prostaglandin synthesis, and produce analgesic, anti-inflammatory, and antipyretic effects. It is widely used in clinical practice but can also cause adverse drug reactions. Common adverse reactions mainly include skin damage, central nervous system damage, respiratory system damage, and gastrointestinal system damage. Studies have found that allergic reactions to nonsteroidal anti-inflammatory drugs are the main cause of Kounis syndrome (3). The article suggests that the mechanism behind the induction of Kounis syndrome by NSAIDs may involve two aspects (4). The first mechanism is related to drug-specific IgE antibodies. The second mechanism involves the inhibition of cyclooxygenase, which stimulates the lipoxygenase pathway of arachidonic acid metabolism, leading to increased production of leukotrienes. The latter is specific to NSAID-induced Kounis syndrome. To the best of our knowledge, this is the first reported case of flurbiprofen allergy-induced Kounis syndrome. The patient in this case was a middle-aged woman with no history of coronary atherosclerotic heart disease, and the initial symptoms were rash and difficulty breathing, accompanied by signs of shock, indicating a serious condition.

Since its first report in 1991, Kounis syndrome has mainly occurred in Southern Europe, with reported incidence rates of 2–19.4 cases per 100,000 inhabitants (5). Currently, there are no reported incidence rates among Asian populations, and only sporadic cases were reported in China. In our patient, coronary angiography did not reveal significant coronary stenosis or acute thrombus formation. Subsequent electrocardiogram showed resolution of ST segment elevation in the inferior leads soon, suggesting coronary artery spasm as the cause, which corresponded to the characteristics of Kounis syndrome Type I. Now, Kounis syndrome is believed to be divided into three types (6). In these three types, patients present with symptoms similar to myocardial infarction, but with varying conditions of the coronary arteries. Type I is characterized by patients having normal coronary arteries, with the coronary artery spasm being caused by an allergic reaction. It is a common cause of non-obstructive myocardial infarction (7). Type II is characterized by patients already having underlying coronary artery atherosclerosis, and the allergic reaction leading to erosion or rupture of the plaque (8). Type III refers to patients who have undergone coronary artery stenting, but experience acute thrombosis due to an allergic reaction. Type III can be further divided into two subtypes, with type IIIa being characterized by thrombus formation within the stent, while type IIIb is characterized by in-stent restenosis (9). Among all Kounis syndrome patients, Type I accounts for 72.6%, while Type II and Type III account for 22.3% and 5.1% (10). The majority of patients (68%) are between 40 and 70 years old, and pediatric cases are relatively rare (11). Males make up 74.3% of the cases.

Kounis syndrome is characterized by symptoms and signs of allergic reaction accompanied by acute coronary syndrome manifestations, such as chest pain, dizziness, nausea, vomiting, difficulty breathing, syncope, palpitations, pallor, hypotension, and bradycardia (9). It is important to note that in patients with shock, a rash may be absent, which increases the risk of misdiagnosis (12). Stress-induced cardiomyopathy should be considered in the differential diagnosis of Kounis syndrome since these two clinical conditions can coexist, forming the so-called “adrenaline-takotsubo-anaphylaxis-Kounis (ATAK complex)”, and allergic myocarditis should also be distinguished (13). New imaging techniques such as dynamic contrast-enhanced cardiac magnetic resonance imaging and myocardial single-photon emission computed tomography can also assist in the diagnosis (14, 15). Additionally, research has also indicated that performing flow cytometry-based proliferation assay *in vitro* can better elucidate the association between drugs and allergic reactions (16). Previous studies have reported that the information provided by electrocardiography may be unreliable. It has been reported that ST segment changes are not consistent with spastic coronary artery discovered by coronary angiography (17). Currently, it is believed that investigating the allergen source and personal medical history is helpful in the diagnosis of Kounis syndrome, with 25.1% of patients having a history of allergies, and the usual allergens needing to be traced back to 1–6 h before the clinical onset (10).

Treating Kounis syndrome presents a challenge as there may be conflicting approaches to cardiovascular and allergic reaction treatments. In this case, aggressive fluid resuscitation can improve hypotension, but it also increases cardiac volume load and poses a risk of precipitating heart failure. Currently, it is believed that anti-allergic treatment can improve symptoms in the majority of patients with Type I Kounis syndrome. In patients with normal blood pressure, most medications, including nitroglycerin, can be used (5). For patients with Type II and Type III Kounis syndrome, treatment should be initiated with an acute coronary event protocol combined with corticosteroids and antihistamines. However, there are some specific considerations when choosing medications. β-blockers for acute coronary artery disease may exacerbate coronary artery spasm. Vasodilators, such as nitrates and calcium channel blockers, can worsen hypotension. Opiates, such as morphine, used for relieving acute chest pain, may worsen allergic reactions, making fentanyl and its derivatives preferable (5). What's more, adrenaline carries the risk of exacerbating ischemia and coronary artery vasospasm. Adrenaline without sulfite, glucagon, and methoxamine may be more effective (18). Additionally, for Type III Kounis syndrome patients, aggressive treatment for acute myocardial infarction and urgent clot aspiration from stenting are advised. Identifying the allergen and intensifying anti-allergic treatment is also crucial. If these measures fail, it seems inevitable to remove the stent (19).

## Conclusions

Kounis syndrome is not uncommon, but it is often underdiagnosed. If the underlying cause is not effectively treated, these patients may experience more coronary events or require more interventional treatments. Therefore, clinicians should pay attention to this specific coronary artery disease related to allergic response.

## Data Availability

The raw data supporting the conclusions of this article will be made available by the authors, without undue reservation.
