# Supplementary material for: Case Report: Flurbiprofen-induced Type I Kounis syndrome
Source: Front Cardiovasc Med. 2023 Nov 20;10:1284408. doi: 10.3389/fcvm.2023.1284408 (PMC10694269; doi:10.3389/fcvm.2023.1284408)
Supplement: Supplementary file 1 [file Datasheet1.pdf]

CARE case report guidelines

CARE Checklist of information to include when writing a case report

(cc) BY-NC-ND

Topic	Item	Checklist item description	Reported on Line
**Title**	1	The diagnosis or intervention of primary focus followed by the words "case report"	Title Page
**Key Words**	2	2 to 5 key words that identify diagnoses or interventions in this case report, including "case report"	Title Page
**Abstract (no references)**	3a	Introduction: What is unique about this case and what does it add to the scientific literature?	Abstract, Background
	3b	Main symptoms and/or important clinical findings	Abstract, Case Summary
	3c	The main diagnoses, therapeutic interventions, and outcomes	Abstract, Case Summary
	3d	Conclusion—What is the main "take-away" lesson(s) from this case?	Abstract, Conclusions
**Introduction**	4	One or two paragraphs summarizing why this case is unique (**may include references**)	Introduction
**Patient Information**	5a	De-identified patient specific information.	Case Presentation, Paragraph 1
	5b	Primary concerns and symptoms of the patient.	Case Presentation, Paragraph 1
	5c	Medical, family, and psycho-social history including relevant genetic information	Case Presentation, Paragraph 1
	5d	Relevant past interventions with outcomes	Case Presentation, Paragraph 1
**Clinical Findings**	6	Describe significant physical examination (PE) and important clinical findings.	Case Presentation, Paragraph 2,3
**Timeline**	7	Historical and current information from this episode of care organized as a timeline	Inapplicable
**Diagnostic Assessment**	8a	Diagnostic testing (such as PE, laboratory testing, imaging, surveys).	Case Presentation, Paragraph 2,3,4
	8b	Diagnostic challenges (such as access to testing, financial, or cultural)	Inapplicable
	8c	Diagnosis (including other diagnoses considered)	Case Presentation, Paragraph 4
	8d	Prognosis (such as staging in oncology) where applicable	Case Presentation, Paragraph 4
**Therapeutic Intervention**	9a	Types of therapeutic intervention (such as pharmacologic, surgical, preventive, self-care)	Case Presentation, Paragraph 2,3,4
	9b	Administration of therapeutic intervention (such as dosage, strength, duration)	Case Presentation, Paragraph 2,3,4
	9c	Changes in therapeutic intervention (with rationale)	Case Presentation, Paragraph 4
**Follow-up and Outcomes**	10a	Clinician and patient-assessed outcomes (if available)	Case Presentation, Paragraph 4
	10b	Important follow-up diagnostic and other test results	Case Presentation, Paragraph 4
	10c	Intervention adherence and tolerability (How was this assessed?)	Case Presentation, Paragraph 4
	10d	Adverse and unanticipated events	None
**Discussion**	11a	A scientific discussion of the strengths AND limitations associated with this case report	Discussion, Paragraph 2
	11b	Discussion of the relevant medical literature **with references**.	Discussion, Paragraph 1,2,3
	11c	The scientific rationale for any conclusions (including assessment of possible causes)	Discussion, Paragraph 1,2,3
	11d	The primary "take-away" lessons of this case report (without references) in a one paragraph conclusion	Conclusions
**Patient Perspective**	12	The patient should share their perspective in one to two paragraphs on the treatment(s) they received	Case Presentation, Paragraph 4
**Informed Consent**	13	Did the patient give informed consent? Please provide if requested	**Yes** ☑ **No** ☐
